# Epidemiology, clinical characteristics, and outcome of enterococcal bloodstream infections in critically ill patients in China: a single-center retrospective 10-year analysis

**DOI:** 10.1128/spectrum.01067-25

**Published:** 2025-08-11

**Authors:** Ye Jin, Zihan Yi, Yue Hu, Man Huang

**Affiliations:** 1 Department of General Intensive Care Unit, the Second Affiliated Hospital of Zhejiang University School of Medicine 89681, Hangzhou, Zhejiang, People's Republic of China; 2 Key Laboratory of Multiple Organ Failure (Zhejiang University), Ministry of Education 12377, Hangzhou, Zhejiang, People's Republic of China; 3 Key Laboratory of Respiratory Disease of Zhejiang Province, Department of Respiratory and Critical Care Medicine, the Second Affiliated Hospital of Zhejiang University School of Medicine 89681, Hangzhou, Zhejiang, People's Republic of China; The University of Texas at Tyler, Tyler, Texas, USA

**Keywords:** bloodstream infection, intensive care unit, *Enterococcus*, mortality, risk factors

## Abstract

Enterococcal bloodstream infections (BSIs) in critically ill patients are linked to multidrug resistance (MDR), high morbidity, and increased mortality. We conducted a retrospective cohort study over a decade at a 3,500-bed teaching hospital, including intensive care unit patients diagnosed with enterococcal BSIs. Data on demographics, comorbidities, severity scores, treatments, and microbiological profiles were analyzed. Risk factors for infection and mortality were assessed using multivariate logistic regression. Among 101 patients, *Enterococcus faecium* was the predominant species, with nearly half of these isolates exhibiting MDR, particularly to ampicillin, aminoglycosides, and fluoroquinolones. Primary infection sources among all patients included abdominal (58.4%), catheter-related (32.7%), and wound infections (10.9%). Key mortality risk factors included a high Charlson comorbidity index (CCI ≥ 5; hazard ratio [HR] = 18.18, *P* < 0.001), male sex (HR = 3.09, *P* = 0.0174), prolonged hospitalization, mechanical ventilation >7 days, central venous catheter (CVC) use, prior broad-spectrum antibiotic exposure, immunosuppression, and delayed antimicrobial therapy (>24 hours; HR = 11.11, *P* < 0.001). Prior fluoroquinolone exposure and inadequate source control also increased mortality risk. In contrast, early source control (HR = 0.08, *P* < 0.001), timely targeted therapy (HR = 0.09, *P* = 0.00214), and early CVC removal (HR = 0.01, *P* < 0.001) improved survival. Persistent bacteremia and inflammatory markers were not independent mortality predictors. Future research should optimize antibiotic duration, refine risk prediction models, and develop novel therapies for MDR *Enterococcus* infections.

**IMPORTANCE:**

Enterococcal bloodstream infections are a serious concern in critically ill patients, often associated with multidrug resistance (MDR) and high mortality rates. This study highlights the importance of early intervention, including source control and timely antimicrobial therapy, in improving patient outcomes. By identifying key risk factors for mortality, such as comorbidities, male sex, and delayed treatment, this research provides valuable insights for clinicians in managing these infections. Notably, the study emphasizes the crucial role of early removal of central venous catheters and targeted therapy in reducing mortality risk. These findings underscore the need for effective infection prevention strategies and timely clinical decisions in critically ill patients. This work lays the foundation for future research aimed at optimizing treatment approaches and combating the growing challenge of MDR *Enterococcus* infections in intensive care settings.

## INTRODUCTION

Bloodstream infections (BSIs) pose a significant threat with an elevated mortality rate of approximately 15% at 30 days and an expected twofold increase within 3 years (1). Among the causative agents, *Enterococcus* species, part of the normal gastrointestinal flora, rank as the third leading cause of nosocomial BSIs in the USA, contributing to 9.4% of all bacteremia cases (2). Consistent with this, a multicenter study in Italy highlighted that *Enterococcus* accounted for 11.4% of all bacteremia cases during a 1 year survey (3). The majority of *Enterococcus* BSI cases stem from urinary tract infections, intra-abdominal infections, device-related infections, and endocarditis (4).

Enterococci are frequent culprits of intensive care unit (ICU)-acquired BSIs (5), yet their clinical significance remains ambiguous. Despite enterococcal BSIs predominantly affecting critically ill patients and being linked to a mortality rate ranging from 23% to 48% (6, 7), many physicians perceive this pathogen to exhibit relatively low virulence. It is widely acknowledged that enterococcal BSIs frequently manifest in patients with underlying illnesses (8), and the associated high mortality is often attributed to these comorbid conditions rather than the intrinsic pathogenicity of *Enterococcus *spp. In contrast to this notion, a study that encompassed patients with enterococcal BSIs and matched uninfected controls revealed a noteworthy excess mortality attributable to *Enterococcus *spp., amounting to 31% (9).

Presently, uncertainties persist regarding whether enterococcal BSIs are the primary cause of death in critically ill patients or merely a clinical indicator associated with a heightened risk of mortality. Some researchers argue that this association may be confounded by the fact that patients with vancomycin-resistant enterococci and vancomycin- and linezolid-resistant enterococci BSIs are often critically ill and present with multiple comorbidities, complicating the accurate assessment of mortality risk (10). To address this gap, we initiated a study with the objective of investigating the epidemiology, clinical characteristics, and outcomes of ICU-acquired enterococcal BSIs in critically ill patients. Additionally, we aimed to estimate the mortality rate associated with this infection from both individual patient and disease perspectives. This comprehensive analysis seeks to provide valuable insights into the clinical implications and impact of *Enterococcus* species in critically ill patients, contributing to enhanced management and preventive strategies in ICU settings.

## MATERIALS AND METHODS

### Study design

This retrospective, observational study focused on ICU patients diagnosed with ICU-acquired enterococcal BSIs at the Second Affiliated Hospital of Zhejiang University, School of Medicine. This 3,500-bed public teaching hospital, situated in east China, accommodates approximately 8,000,000 patients annually. The Department of Intensive Care Medicine comprises nine multidisciplinary ICUs with a combined total of 178 ICU beds. These units cater to postoperative care following cardiac, neurosurgery, trauma-related, emergency, vascular, burn, thoracic, respiratory, and visceral surgery. Each unit handles mixed patient populations, encompassing both medical and surgical cases. The study included critically ill patients with positive blood cultures for *Enterococcus *spp., treated between January 2014 and December 2023. Data were retrospectively extracted from the hospital’s electronic medical record system. The extracted data included patients’ demographic information (age, sex), clinical characteristics (underlying diseases, comorbidities), laboratory test results, microbiological profiles (including bacterial species identification and antimicrobial susceptibility), details of antimicrobial therapies administered (timing, type, duration), severity scores such as Acute Physiology and Chronic Health Evaluation II (APACHE II) score, details of invasive procedures (e.g., central venous catheter [CVC] use, mechanical ventilation), and clinical outcomes (mortality, length of ICU stay). The use of a large, representative sample from a single, well-established tertiary teaching hospital provides a robust data set, enabling comprehensive analysis of risk factors and outcomes in critically ill patients with enterococcal bloodstream infections.

In adherence to stringent confidentiality mandates, clinical data were meticulously retrieved from patients’ healthcare records, subsequently subjected to a rigorous anonymization process within the database.

### Study population

Patients were eligible for inclusion if they met all the following criteria:

- Age ≥ 18 years
- Admitted to the ICU.
- Presence of at least one positive blood culture for *Enterococcus* spp., with concurrent clinical signs of systemic infection.
- Administration of antimicrobial therapy for enterococcal BSIs.

Patients were excluded if they met any of the following conditions:

- Polymicrobial bloodstream infections where *Enterococcus* was not the dominant pathogen.
- Patients with incomplete medical records or missing microbiological data.
- Patients receiving palliative care only or those with do-not-resuscitate orders.

### Definitions and classification of infection sources

ICU-acquired enterococcal BSI was defined as the first occurrence of a positive blood culture at least 48 hours after ICU admission, with no prior positive cultures for the same pathogen in the preceding 30 days. The source of bacteremia was determined through *post hoc* physician assessment based on predefined criteria, incorporating microbiological, radiological, and clinical findings. Infections were classified as follows:

- Catheter-related bloodstream infection: defined according to the Infectious Diseases Society of America criteria (11) requiring either (i) simultaneous positive blood cultures from a peripheral vein and the catheter tip or (ii) differential time-to-positivity of ≥2 hours between peripheral and catheter-drawn cultures.
- Abdominal-source infection: diagnosed based on positive intra-abdominal culture results and imaging findings indicative of intra-abdominal sepsis.
- Urinary tract infection-associated bacteremia: defined as a positive urine culture with significant bacteriuria (>10⁵ CFU/mL) in conjunction with symptoms of urinary tract infection.
- Primary bacteremia (no identifiable source): cases in which no specific infection focus could be identified after a comprehensive diagnostic workup.
- Appropriate therapy: as the administration of at least one antimicrobial agent to which the *Enterococcus* isolate was susceptible *in vitro*, initiated within 48 hours of the first positive blood culture.

### Microbiological analysis and antimicrobial susceptibility testing

Blood cultures were processed using the BacT/Alert automated system (Organon Teknika, Durham, NC, USA). Bacterial identification was performed using matrix-assisted laser desorption/ionization time-of-flight mass spectrometry.

Antimicrobial susceptibility testing was conducted according to the Clinical and Laboratory Standards Institute guidelines. The tested antibiotics included penicillin, ampicillin, high-dose gentamicin (120 mg/mL), high-dose streptomycin, ciprofloxacin, levofloxacin, erythromycin, quinupristin/dalfopristin, linezolid, vancomycin, tetracycline, tigecycline, and nitrofurantoin. The antibiotics tested were selected based on their clinical relevance in treating *Enterococcus* infections and their representation in international and local empirical treatment guidelines. These included first-line agents for susceptible strains (e.g., penicillin, ampicillin), synergistic agents used in combination therapy for serious infections (e.g., high-dose gentamicin and streptomycin), fluoroquinolones (ciprofloxacin, levofloxacin), macrolides (erythromycin), and last-resort or alternative agents for multidrug-resistant isolates (e.g., linezolid, vancomycin, tigecycline, quinupristin/dalfopristin, and nitrofurantoin). This comprehensive panel ensures adequate assessment of resistance patterns across both commonly used and reserve antibiotics in critically ill patients.

### Data collection

The demographic and clinical data were retrieved from the electronic patient data management system, including variables such as age, gender, admission diagnosis, comorbidities, details of antimicrobial therapy, clinical outcomes, Sequential Organ Failure Assessment (SOFA) scores concurrent with positive cultures, occurrences of septic shock, laboratory test results, and other pertinent parameters. Furthermore, details regarding the utilization of indwelling catheters, administered antibiotics, dosage, and treatment duration were documented. The severity of each patient’s condition was evaluated by computing the APACHE II score upon admission to the ICU.

### Statistical analysis

Continuous variables were presented as mean ± standard deviation (SD) or median with interquartile range (IQR), depending on the data distribution, while categorical variables were expressed as frequencies and percentages. Comparisons between groups were conducted using the Student’s *t*-test or Mann-Whitney U-test for continuous variables and the χ^2^ test or Fisher’s exact test for categorical variables, as appropriate. To rank the clinical predictors associated with mortality in enterococcal BSI, a random forest prediction model was employed to compute the permutation accuracy importance metric (12, 13). To mitigate potential confounding effects, propensity score matching (1:1 matching) was employed to compare clinical outcomes between treatment groups, with matching variables including age, SOFA score, APACHE II score, and infection source. A subgroup analysis was conducted, excluding patients who died within 48 hours of bacteremia onset, to assess the robustness of the findings. All statistical analyses were conducted using SPSS (version 28), and a *P*-value <0.05 was considered statistically significant.

## RESULTS

### Patient characteristics

The baseline demographic and clinical characteristics of patients with enterococcal BSIs are summarized in Table 1. The median age of the cohort was 62 years, with a substantial proportion (43.2%) being older than 65 years. Males accounted for 48.5% (*n* = 49) of the study population. Most patients (98.8%) had at least one comorbidity, with a median Charlson comorbidity index (CCI) score of 5 (IQR: 4–6). Hypertension (44.6%), diabetes (36.7%), malignancy (20.8%), atrial fibrillation (20.8%), and chronic obstructive pulmonary disease (6.9%) were the most prevalent comorbid conditions. Notably, 10.9% of patients had a history of ICU admission within the preceding 3 months.

**TABLE 1:** Baseline demographic and clinical characteristics, and admission diagnosis in critically ill patients with enterococcal BSIs

Parameter/category	Total patients	Survivors	Non-survivors	*P*-value
	(*n* = 101)	(*n* = 58)	(*n* = 43)	
Male sex (*n*)	70	35	35	0.023
Age, median years (range)	62 ± 17	64 ± 16	60 ± 18	0.297
Age ≥65 years (*n*)	49	28	21	0.955
Comorbid conditions on admission (*n*, %)				
Coronary heart disease	8 (7.9)	5	3	0.762
Diabetes mellitus	37 (36.7)	23	14	0.822
COPD^*a*^	7 (6.9)	2	5	0.110
Liver cirrhosis	3 (3.0)	2	1	0.742
Chronic kidney disease	2 (2.0)	1	1	0.830
Hypertension	45 (44.6)	23	22	0.250
Atrial fibrillation	21 (20.8)	13	8	0.641
Hematologic malignancy	3 (3.0)	0	3	0.041
Solid tumor	21 (20.8)	12	9	0.976
Implantable cardiac devices	3 (3.0)	1	2	0.392
Stem cell transplantation	1 (1)	0	1	0.243
AIDS	0	0	0	N/A^*b*^
Organ transplantation	3 (3.0)	2	1	0.742
Admission diagnosis (*n*, %)				
Internal medicine	84 (83.2)	47	37	0.506
Abdominal surgery	17 (16.8)	9	8	0.682
Surgery other than abdominal	24 (23.8)	18	6	0.046
Infectious diseases	40 (39.6)	20	20	0.222
Previous ICU admission	11 (10.9)	6	5	0.838

^*a*^ Chronic obstructive pulmonary disease.

^*b*^ N/A indicates not applicable.

No significant differences were observed in demographic characteristics between survivors and non-survivors. However, non-survivors exhibited a higher burden of chronic comorbidities (median CCI score: 6 vs 3; *P *< 0.001) and were more frequently affected by cirrhosis and septic shock. Additionally, non-survivors presented with significantly higher disease severity at baseline, as reflected by higher APACHE II scores (median: 22 vs 18; *P *< 0.001). These patients also had greater requirements for extracorporeal organ support both before and after enterococcal BSI onset, longer durations of CVC placement, and extended ICU and hospital stays (Table 2).

**TABLE 2:** Patient characteristics at the day of intensive care unit-acquired BSIs

Parameter/category	Total patients (*n* = 101)	Survivors (*n* = 58)	Non-survivors (*n* = 43)	*P*-value
ICU stay (mean, days)	15	15	17	0.154
APACHE II score	20	18	22	0.001
SOFA	7 ± 4	6 ± 3	9 ± 4	0.038
Charlson comorbidity score	4	3	6	0.022
RRT^*a*^	26	8	18	0.001
Mechanical ventilation	91	49	42	0.028
Blood transfusion	47	18	29	0.000
ECMO^*b*^ support	3	3	0	0.130
Enteral nutrition	54	36	18	0.001
Surgery	82	58	24	0.001
Indwelling catheters				
Arterial	89	51	38	0.946
Pulmonary artery	0	0	0	N/A^*e*^
Nasogastric tube	94	53	41	0.437
Central venous catheter (CVC)	76	42	34	0.443
Other types	101	58	43	N/A
Most likely source of bacteremia				
Pulmonary	5	2	3	0.658
Catheter infection	33	19	14	0.983
Abdominal	59	30	29	0.113
Urinary tract	2	1	1	0.830
Endocarditis	0	0	0	N/A
Wound	11	6	5	0.838
Others	9	4	5	0.409
Unknown	6	5	1	0.186
Inflammatory parameters^*a*^				
WBC^*c*^ count (10^9^/L)	10.1	9.6	10.5	0.526
Procalcitonin (µg/L)	0.56	0.41	0.85	0.329
CRP^*d*^ (mg/dL)	83.5	84.5	83.5	0.381
Lactate (mmol/L)	1.6	1.5	1.6	0.023
≥3 consecutive positive blood cultures	18	8	2	0.219

^*a*^ RRT, renal replacement therapy.

^*b*^ Extracorporeal membrane oxygenation.

^*c*^ WBC, white blood cell.

^*d*^ CRP, C-reactive protein.

^*e*^ N/A indicates not applicable.

As shown in Table 2, the predominant sources of bacteremia were abdominal infections (59 cases, 58.4%), catheter-related infections (33 cases, 32.7%), and wound infections (11 cases, 10.9%), with no significant differences between survivors and non-survivors. Although indwelling catheters, including arterial catheters, nasogastric tubes, and CVCs, were present in nearly all patients, their presence alone was not associated with increased mortality, suggesting that host factors and disease severity played a more critical role in determining patient outcomes.

Among metabolic biomarkers, elevated serum lactate levels were significantly associated with mortality (*P* = 0.023), reinforcing its prognostic value in critically ill patients (14). In contrast, systemic inflammatory markers, including white blood cell count, C-reactive protein, and procalcitonin, did not significantly differ between groups, indicating that traditional inflammatory markers alone were insufficient predictors of mortality in this cohort. Persistent bacteremia (≥3 consecutive positive blood cultures) was not significantly associated with mortality (17.8% overall; *P* = 0.219), suggesting that persistent infection was not an independent determinant of prognosis.

### Species distribution and antimicrobial susceptibility of isolates

Microbiological analysis identified five distinct genera of *Enterococcus* within this cohort. The most prevalent species was *Enterococcus faecium* (*n* = 59, 58.4%), succeeded by *Enterococcus faecalis* (*n* = 27, 26.7%), *Enterococcus avium* (*n* = 6, 5.9%), *Enterococcus gallinarum* (*n* = 7, 6.9%), and *Enterococcus casseliflavus* (*n* = 2, 2.0%). Antimicrobial susceptibility patterns varied significantly across species, with *E. faecium* demonstrating the highest resistance rates to multiple drug classes (Table 3), including beta-lactams (ampicillin), aminoglycosides (high-dose gentamicin and streptomycin), and fluoroquinolones (ciprofloxacin and levofloxacin).

**TABLE 3:** Species distribution and antimicrobial susceptibility of *Enterococcus* isolates^*a*^

Antimicrobial agent	*E. faecium* (*n* = 59)	*E. faecalis* (*n* = 27)	*E. avium* (*n* = 6)	*E. gallinarum* (*n* = 7)	*E. casseliflavus* (*n* = 2)	Total (*n* = 101)
Penicillin S (*n*, %)	1 (1.7)	22 (81.5)	2 (33.3)	4 (57.1)	1 (50.0)	30 (29.7)
Ampicillin S (*n*, %)	1 (1.7)	22 (81.5)	3 (50.0)	1 (14.3)	1 (50.0)	28 (27.7)
High-level gentamicin S (*n*, %)	27 (45.8)	13 (48.1)	3 (50.0)	7 (100.0)	1 (50.0)	51 (50.5)
High-level streptomycin S (*n*, %)	14 (23.7)	19 (70.4)	4 (66.7)	2 (28.6)	0	39 (38.6)
Ciprofloxacin S (*n*, %)	11 (18.6)	4 (14.8)	5 (83.3)	5 (71.4)	0	25 (24.6)
Levofloxacin S (*n*, %)	12 (20.3)	3 (11.1)	5 (83.3)	5 (71.4)	0	25 (24.6)
Erythromycin S (*n*, %)	1 (1.7)	3 (11.1)	1 (16.7)	1 (14.3)	0	6 (5.9)
Quinupristin/dalfopristin S (*n*, %)	50 (84.7)	3 (11.1)	0	6 (85.7)	2 (100.0)	61 (60.4)
Vancomycin S (*n*, %)	59 (100.0)	27 (100.0)	6 (100.0)	0	0	92 (91.1)
Tigecycline S (*n*, %)	59 (100.0)	27 (100.0)	6 (100.0)	4 (57.1)	2 (100.0)	98 (97.0)
Tetracycline S (*n*, %)	39 (66.1)	3 (11.1)	0	6 (85.7)	2 (100.0)	47 (46.5)
Nitrofurantoin S (*n*, %)	9 (15.3)	24 (88.9)	6 (100.0)	7 (100.0)	2 (100.0)	48 (47.5)
MDR (*n*, %)	34 (57.6)	5 (18.5)	3 (50.0)	2 (28.6)	2 (100.0)	46 (45.5)

^*a*^ Abbreviations: S, susceptible; MDR, multidrug resistance (resistance to ≥3 antimicrobial classes).

Overall, the susceptibility rates to penicillin and ampicillin were 29.7% and 27.7%, respectively. *E. faecium* exhibited the highest level of resistance, with only 1.7% of isolates susceptible to either agent. In contrast, *E. faecalis* remained largely susceptible, with 81.5% of isolates retaining susceptibility to both penicillin and ampicillin (*P* < 0.001). Non-faecium species demonstrated variable susceptibility, with *E. avium* (50.0%) and *E. gallinarum* (14.3%) exhibiting moderate resistance to ampicillin.

High-level aminoglycoside resistance was assessed for gentamicin and streptomycin, revealing a susceptibility rate of 50.5% and 38.6%, respectively. *E. faecium* exhibited significantly higher streptomycin resistance compared to *E. faecalis* (76.3% vs 29.6%, *P* < 0.001), whereas high-level gentamicin resistance was more evenly distributed between these species (54.2% vs 51.9%, *P* = 0.673).

Fluoroquinolone resistance was widespread, with susceptibility rates of only 24.6% for both ciprofloxacin and levofloxacin. However, *E. avium* and *E. gallinarum* exhibited significantly higher susceptibility to fluoroquinolones (83.3% and 71.4%, respectively) compared to *E. faecium* and *E. faecalis* (*P* < 0.001). Similarly, erythromycin resistance was highly prevalent (94.1%), with only 5.9% of isolates remaining susceptible, limiting the clinical utility of macrolides in enterococcal BSI treatment.

All *E. faecium*, *E. faecalis,* and *E. avium* isolates remained susceptible to vancomycin, whereas *E. gallinarum* and *E. casseliflavus* exhibited intrinsic resistance (15). Tigecycline demonstrated the highest efficacy, with an overall susceptibility rate of 97.0%. Quinupristin/dalfopristin demonstrated potent *in vitro* activity against *E. faecium*, as well as *E. gallinarum* and *E. casseliflavus*, with susceptibility rates of 84.7%, 85.7%, and 100%, respectively.

Tetracycline susceptibility was observed in 46.5% of isolates, with *E. faecium* showing a significantly higher susceptibility rate (66.1%) compared to *E. faecalis* (11.1%). Nitrofurantoin exhibited moderate overall activity (47.5% susceptibility), with *E. faecalis* (88.9%) and non-faecium species (100%) displaying high susceptibility rates, while *E. faecium* demonstrated the highest resistance (84.7%).

Multidrug resistance (MDR), defined as resistance to ≥3 antimicrobial classes, was observed in 45.5% of isolates. *E. faecium* exhibited the highest MDR prevalence (57.6%), followed by *E. casseliflavus* (100.0%) and *E. avium* (50.0%), whereas *E. faecalis* had the lowest MDR rate (18.5%) (*P* < 0.001).

### Clinical management in critically ill patients with ICU-acquired enterococcal BSIs

The management of ICU-acquired enterococcal BSIs remains a significant challenge due to the high prevalence of multidrug resistance and the critically ill status of affected patients. In our cohort, empirical antimicrobial therapy was initiated in 96.0% of cases, with vancomycin and linezolid being the most frequently administered agents (Table 4). Following susceptibility testing, targeted therapy was implemented in 83.2% of patients, with daptomycin and linezolid as the most utilized definitive treatments. The median duration of antibiotic therapy was 7 days (IQR: 7–14 days). The median duration of antibiotic treatment was significantly shorter in non-survivors (5 ± 4 days vs 8 ± 8 days, *P* = 0.041). This discrepancy is likely attributable to early mortality in non-survivors before completion of therapy rather than differences in treatment strategy.

**TABLE 4:** Clinical management in critically ill patients with ICU-acquired enterococcal BSIs

Clinical management	Total patients (*n* = 101)	Survivors (*n* = 58)	Non-survivors (*n* = 43)	*P*-value
Antimicrobial strategy (*n*)				
Preventive^*a*^	4	3	1	0.468
Empirical^*b*^	97	55	42	0.468
Targeted^*c*^	84	68	16	0.898
Delayed^*d*^	18	10	8	N/A^*g*^
None	0	0	0	N/A
Received appropriate therapy within 24 hours	23	20	3	0.0001
Duration of antibiotic treatment^*e*^ (mean ± SD, *n* = 81)	7 ± 7 (81)	8 ± 8	5 ± 4	N/A
Prior antibiotic exposure during ICU stays (*n*)				
Vancomycin	12	5	7	0.240
Linezolid	9	4	5	0.409
Cephalosporin	28	17	11	0.679
Carbapenems	45	22	23	0.120
Fluoroquinolones	13	4	9	0.037
Cephalosporin/beta-lactamase inhibitor combination	44	21	23	0.083
Others	48	25	23	0.301
Number (%) of indwelling catheters that were removed within 3 days				
Peripheral arterial catheter	0	0	0	N/A
CVC	63	57	6	0.0001
RRT catheter	1	0	1	0.243
CVC removal timing (*n*)				
Early removal (≤48 h)	40	35	5	N/A
Late removal (>48 h)	23	15	8	N/A
Duration of bacteremia, median (IQR), days				
Early CVC removal group	6	N/A	N/A	N/A
Late CVC removal group	13	N/A	N/A	N/A
Intra-abdominal infection drainage (*n*)				
Adequate drainage^*f*^	38	33	5	0.001
Inadequate or no drainage group	53	29	24	0.001

^*a*^ Preventive treatment: administering antibiotic prophylaxis to asymptomatic patients.

^*b*^ Empirical therapy: administering empiric treatment to febrile patients or patients with elevated inflammatory markers in the blood, despite negative bacterial cultures.

^*c*^ Targeted therapy was initiated within 24 hours of the identification of positive cultures.

^*d*^ Delayed: antimicrobial therapy was initiated more than 24 hours after the identification of positive cultures.

^*e*^ Duration of antibiotic treatment: the duration of antibiotic treatment was only calculated for patients for whom treatment was discontinued before the last day in the ICU, as there was no record of antibiotic treatment after ICU discharge.

^*f*^ Adequate abdominal drainage was defined as timely and effective source control of intra-abdominal infections, as determined by either radiological or surgical intervention, with documentation of successful fluid or abscess evacuation in the medical records. Timeliness was defined as intervention performed within 48 hours of bacteremia diagnosis. Inadequate drainage referred to either absence of intervention or documented failure of source control (e.g., residual abscess or persistent signs of sepsis despite intervention).

^*g*^ N/A indicates not applicable.

The timing of appropriate antimicrobial therapy was a critical determinant of clinical outcomes. Patients who received appropriate therapy within 24 hours had a significantly lower mortality rate compared to those with delayed treatment initiation (13.0% vs 44.4%, *P* = 0.0001). In contrast, patients who experienced treatment delays were more likely to require salvage therapies, including combination antimicrobial regimens and adjunctive interventions such as source control and extracorporeal organ support. These findings underscore the importance of early pathogen-directed therapy in improving survival rates in critically ill patients with enterococcal BSI.

A substantial proportion of patients had prior exposure to broad-spectrum antibiotics during their ICU stay, which may have influenced pathogen selection and treatment response. The most frequently used antibiotic classes were carbapenems (45/101, 44.6%) and cephalosporin/beta-lactamase inhibitor combinations (44/101, 43.6%), though their association with mortality was not statistically significant (*P* = 0.120 and *P* = 0.083, respectively). However, prior fluoroquinolone exposure was significantly associated with increased mortality (nine vs four cases, *P* = 0.037). This finding suggests that fluoroquinolone use may contribute to enterococcal resistance and worse clinical outcomes (16).

Source control played a pivotal role in the management of enterococcal BSI, particularly through the removal of CVCs. Among patients with CVC-associated infections, early CVC removal (within 48 hours of BSI diagnosis) was significantly associated with a shorter duration of bacteremia (median 6 days vs 13 days, *P* < 0.001). Additionally, surgical intervention for intra-abdominal infections was required in 23.3% of cases, emphasizing the importance of timely surgical source control. As shown in Table 4, in patients with undrained intra-abdominal infections, mortality was significantly higher compared to those who underwent adequate drainage (29/53, 45.3% vs 5/38, 13.2%, *P* < 0.001).

### Predictors of mortality and the impact of early interventions in ICU-acquired enterococcal BSIs

**Fig 1:**
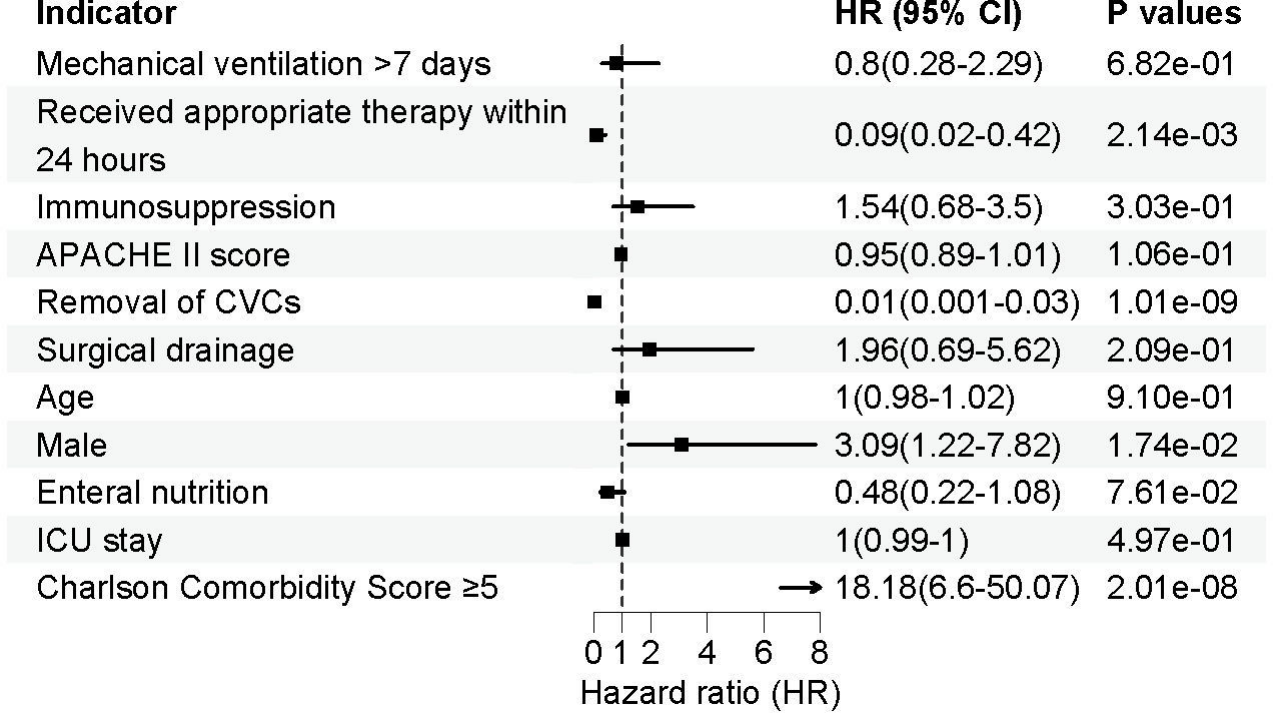
Forest plot of multivariate Cox proportional hazards analysis for predictors of mortality in ICU patients with enterococcal bloodstream infections. Significant protective factors include the administration of early appropriate antimicrobial therapy within 24 hours (HR = 0.09, 95% CI: 0.02–0.42, *P* = 0.00214) and the early removal of CVCs (HR = 0.01, 95% CI: 0.001–0.03, *P* = 1.01e−09). In contrast, male sex (HR = 3.09, 95% CI: 1.22–7.82, *P* = 0.0174) and a CCI ≥5 (HR = 18.18, 95% CI: 6.6–50.07, *P* = 2.01e−08) were identified as significant risk factors for increased mortality. Other variables, such as age, APACHE II score, ICU length of stay, immunosuppression, and mechanical ventilation exceeding 7 days, were not independently associated with mortality. The dashed vertical line represents the null value (HR = 1), indicating no effect.

To identify independent risk factors associated with ICU-acquired enterococcal BSI, we conducted a multivariable Cox regression analysis incorporating key demographic, clinical, and treatment-related variables. As shown in Fig. 1, a Charlson comorbidity score ≥5 was strongly associated with an increased risk of poor prognosis (HR = 18.18, 95% CI: 6.6–50.07, *P* = 2.01 × 10^−^⁸), underscoring the impact of preexisting comorbidities on patient outcomes. Additionally, male sex was identified as an independent risk factor for adverse outcomes (HR = 3.09, 95% CI: 1.22–7.82, *P* = 0.0174), suggesting potential biological or healthcare-related disparities that warrant further investigation.

In contrast, early receipt of appropriate antimicrobial therapy within 24 hours was significantly associated with improved survival, demonstrating a 91% reduction in mortality risk (HR = 0.09, 95% CI: 0.02–0.42, *P* = 0.00214). This finding highlights the critical importance of prompt infection management in ICU patients. Furthermore, removal of CVCs emerged as a key protective factor, with a substantial reduction in mortality risk (HR = 0.01, 95% CI: 0.001–0.03, *P* = 1.01 × 10^−^⁹), reinforcing the role of early source control in optimizing patient outcomes.

Other variables, including mechanical ventilation >7 days, immunosuppression, APACHE II score, age, enteral nutrition, and ICU length of stay, did not reach statistical significance. While these factors have been implicated in infection-related outcomes in previous studies, their lack of association in our cohort may reflect context-dependent variability, potentially influenced by confounding variables or the limitations of a single-center study with region-specific patient characteristics and clinical practices.

## DISCUSSION

Enterococcal BSIs in critically ill patients are associated with high morbidity, MDR, and elevated mortality. This 10-year retrospective study provides comprehensive data on ICU-acquired enterococcal BSIs in a major tertiary hospital in China, highlighting the influence of host factors, invasive procedures, and antimicrobial resistance on patient outcomes.

In this study, we found that a Charlson comorbidity score ≥5 was a strong independent predictor of poor prognosis in ICU-acquired enterococcal BSIs, with an 18-fold increase in mortality risk, emphasizing that critically ill patients with multiple chronic diseases have a markedly increased susceptibility to infection-related complications and adverse prognoses (17, 18). The presence of severe comorbidities may compromise immune function, impair physiological reserves, and prolong hospital stays, thereby facilitating the development and persistence of bloodstream infections. Additionally, male sex was identified as an independent risk factor for mortality in our cohort. Although sex-based differences in sepsis and bloodstream infections remain an area of ongoing research, prior studies have suggested that hormonal influences, immune response variability, and differences in healthcare-seeking behaviors may contribute to the observed disparity in outcomes between male and female patients (19). Further investigation into sex-specific immune mechanisms and antimicrobial treatment responses in enterococcal BSI may provide insights into targeted management strategies.

Our data highlight prolonged CVC use, mechanical ventilation exceeding 7 days, prior broad-spectrum antibiotic exposure, and immunosuppression as independent risk factors. These factors reinforce the role of gut microbiota dysbiosis and invasive medical procedures in facilitating bloodstream invasion (20, 21). Several mechanisms contribute to the pathogenesis of enterococcal BSI, with gastrointestinal translocation serving as a key route, particularly in patients with disrupted gut barrier integrity. Various conditions, including hyperglycemia, ischemia, epigenetic modifications, and the use of specific medications, exacerbate gut barrier dysfunction and increase susceptibility to *Enterococcus* translocation and subsequent bacteremia (21).

In critically ill patients, conditions such as severe trauma, burns, major surgeries, hemorrhagic shock, or severe acute pancreatitis further promote pathological translocation, leading to sepsis and multiple organ dysfunction syndrome, which remain major causes of mortality (22). The significant association between prior *Enterococcus* colonization and subsequent BSI further suggests that gastrointestinal translocation plays a crucial role in bloodstream invasion, particularly in patients exposed to prolonged antibiotic treatment.

Our study also identified *E. faecium* as the predominant species and exhibited the highest MDR rates. Nearly half of all isolates were resistant to ≥3 antibiotic classes. In contrast, *E. faecalis* retained greater susceptibility to beta-lactams and showed lower MDR prevalence. Traditionally, the treatment of *Enterococcus*-related infections has relied on antibiotic regimens combining cell wall inhibitors and aminoglycosides to achieve synergistic bactericidal activity (23). However, *Enterococcus* exhibits an inherent robustness, displaying an unusual capacity to develop resistance to multiple drugs, including macrolides, beta-lactams, tetracyclines, aminoglycosides, and fluoroquinolones (6). This extensive resistance profile significantly complicates treatment, as *Enterococcus* strains often display either intrinsic resistance or reduced susceptibility to many conventional antibiotics. As a result, managing *Enterococcus* infections has become an increasingly formidable challenge, necessitating the development of novel therapeutic strategies and more stringent antimicrobial stewardship to curb the spread of resistant strains. Early and appropriate antimicrobial therapy is essential for improving survival in ICU-acquired enterococcal BSIs. In our cohort, empirical therapy was initiated in 96.0% of cases, with vancomycin and linezolid being the most frequently administered agents. After susceptibility testing, targeted therapy was implemented in 83.2% of patients, with daptomycin and linezolid as the preferred definitive treatments. The timing of appropriate therapy was a key determinant of clinical outcomes, as patients who received effective antimicrobial treatment within 24 hours had a significantly lower mortality rate than those with delayed therapy (13.0% vs 44.4%, *P *< 0.0001). Importantly, excessive antibiotic use did not correlate with improved survival, highlighting the need for balanced antimicrobial stewardship. Prior exposure to fluoroquinolones was significantly associated with increased mortality, suggesting that fluoroquinolone use may contribute to enterococcal resistance and worse clinical outcomes (16).

The low rate of urinary tract-associated bacteremia in our study may reflect the predominance of critically ill ICU patients, in whom catheter-related and intra-abdominal sources are more common. Additionally, diagnostic challenges, such as overlapping symptoms, empirical antibiotic use, and limited documentation, may have led to under-recognition of urinary sources. These results underscore the need for careful source identification and may reflect differences in patient populations or hospital practices across studies. In catheter-related infections, early removal of CVCs was associated with a significantly shorter duration of bacteremia (median: 6 vs 13 days, *P* < 0.001). Similarly, prompt surgical intervention for intra-abdominal infections markedly improved survival outcomes, with patients who did not undergo drainage experiencing a significantly higher mortality rate (45.3% vs 13.2%, *P* < 0.001). However, clinical practice often deviates from these recommendations. In our study, only 62.8% of CVCs were removed within 3 days. These deviations may stem from the perception of *Enterococcus* as a contaminant or a pathogen of low virulence, which may lead to its clinical significance being underestimated and suboptimal management decisions.

Despite appropriate adjustments for confounding factors, the risk of mortality associated with enterococcal BSIs remained substantial, potentially attributable to delayed or insufficient clinical interventions. The mortality rate observed in our study is consistent with ICU bloodstream infection mortality rates, which range from 35% to 50% (5). Notably, recent meta-analyses suggest that shorter antibiotic courses may be as effective as longer regimens in achieving both microbiological and clinical cure in critically ill patients (24). A large randomized controlled trial on complicated intra-abdominal infections further demonstrated that a fixed 4-day antibiotic regimen was non-inferior to prolonged treatment durations (25). However, it remains possible that certain subpopulations within our cohort could derive greater benefit from extended antimicrobial therapy, highlighting the need for individualized treatment strategies based on infection severity, host factors, and microbial resistance patterns.

Our study has several limitations that should be acknowledged. First, its retrospective design inherently carries a risk of selection bias, potentially influencing the interpretation of our findings. Second, as a single-center study, the generalizability of our results to other ICU populations, particularly those with different patient demographics, healthcare practices, and antimicrobial resistance patterns, may be limited. Additionally, while we identified key clinical predictors of mortality, our analysis did not account for host immune response dynamics or bacterial virulence factors, both of which play crucial roles in infection progression and patient outcomes. Finally, molecular typing and detailed genomic analyses of the *Enterococcus* isolates were not performed, which restricts insights into transmission dynamics and resistance mechanisms. Future prospective, multicenter studies incorporating molecular epidemiology, host-pathogen interactions, and virulence determinants are warranted to validate and expand upon our findings, refine risk stratification models, and improve the clinical management of *Enterococcus* bloodstream infections in critically ill patients.

In conclusion, ICU-acquired enterococcal BSIs are associated with high rates of MDR, prolonged hospitalization, and increased mortality risk, particularly when antibiotic therapy is delayed or inadequate. The findings emphasize the importance of early source control, judicious antimicrobial stewardship, and enhanced infection prevention strategies to mitigate the burden of these infections. Future research should focus on novel therapeutic approaches, optimized antibiotic duration, and predictive risk models to improve clinical outcomes in ICU patients with enterococcal BSI. As MDR *Enterococcus* continues to pose a growing threat, ongoing efforts to refine treatment protocols, enhance infection prevention measures, and explore novel therapeutic avenues will be critical in combating this emerging public health challenge.

## Data Availability

The data sets used and/or analyzed during the current study are available from the corresponding author on reasonable request.
